# Young Children of Mothers with a History of Depression Show Attention Bias to Sad Faces: An Eye-tracking Study

**DOI:** 10.1007/s10802-024-01205-w

**Published:** 2024-05-07

**Authors:** Xiaoxue Fu, Scout H. Bolton, Michele Morningstar, Whitney I. Mattson, Xin Feng, Eric E. Nelson

**Affiliations:** 1 Department of Psychology, University of South Carolina, 29201 Columbia, SC USA; 2 Department of Psychological Sciences, University of Missouri, Columbia, MO USA; 3 Department of Psychology, Queen’s University, Kingston, ON Canada; 4 Center for Biobehavioral Health, Abigail Wexner Research Institute, Nationwide Children’s Hospital, Columbus, OH USA; 5 Department of Human and Family Sciences, Ohio State University, Columbus, OH USA; 6 Department of Pediatrics, Ohio State University, Columbus, OH USA

**Keywords:** Attention bias, Maternal depression, Eye tracking, Visual search

## Abstract

Maternal depression is a predictor of the emergence of depression in the offspring. Attention bias (AB) to negative emotional stimuli in children may serve as a risk factor for children of depressed parents. The present study aimed to examine the effect of maternal major depressive disorder (MDD) history on AB to emotional faces in children at age four, before the age of onset for full-blown psychiatric symptoms. The study also compared AB patterns between mothers and their offspring. Fifty-eight mothers and their four-year-old children participated in this study, of which 27 high-risk (HR) children had mothers with MDD during their children’s lifetime. Attention to emotional faces was measured in both children and their mothers using an eye-tracking visual search task. HR children exhibited faster detection and longer dwell time toward the sad than happy target faces. The low-risk (LR) children also displayed a sad bias but to a lesser degree. Children across both groups showed AB towards angry target faces, likely reflecting a normative AB pattern. Our findings indicate that AB to sad faces may serve as an early marker of depression risk. However, we provided limited support for the mother-child association of AB. Future research is needed to examine the longitudinal intergenerational transmission of AB related to depression and possible mechanisms underlying the emergence of AB in offspring of depressed parents.

**Supplementary Information:**

The online version contains supplementary material available at 10.1007/s10802-024-01205-w.

## Introduction

Parental depression is a robust risk factor for the emergence of depression in children. Offspring of depressed mothers are about 3 times more likely to develop major depressive disorder (MDD) than children who experienced no maternal depression (Weissman et al., 2016). Information-processing biases may interact with genetic and environmental factors to potentiate the emergence of depression in the offspring (Gotlib et al., 2020). The existing literature has underscored the association between exposure to maternal depression and attention bias (AB), characterized as preferential attention toward, and/or difficulty disengaging attention from, emotional stimuli (e.g., sad faces) in the offspring (Joormann, 2009). However, the existing literature leaves some important questions unanswered. Most of the studies focused on middle childhood to adolescence, when early symptoms of depression may already be present (Avenevoli et al., 2008; Kessler et al., 2005). Hence, the findings cannot speak to whether AB is present in early childhood before the onset of clinical-level depressive symptoms. Further, the possible role of maternal attentional behavior in modeling or directing the development of AB in children remains unclear. The present study aims to investigate (1) whether young children of mothers with a history of MDD exhibit AB to negative emotional faces compared to offspring of never depressed mothers, and (2) whether mothers with a history of MDD also show AB to negative emotional faces relative to never-depressed mothers. Additionally, we will also explore whether there is an association between mothers and their children’s attention patterns. Understanding the attention patterns associated with depression risk is particularly important for identifying potential targets for early preventative interventions (Browning et al., 2012).

AB to depression-relevant stimuli might be an early-emerging risk factor for depression. Cognitive theories and empirical evidence in adults suggest that depression is associated with increased attention allocation to dysphoric or sad stimuli and reduced attention allocation to happy information (Armstrong & Olatunji, 2012; Suslow et al., 2020). AB toward sad faces prospectively predicted depressive symptoms (Beevers et al., 2011), and training attention away from dysphoric images reduced depression (Wells & Beevers, 2010). Hence, AB potentially plays a causal role in the development of depression. Extending from adult literature, maternal depression may increase psychiatric risk in offspring through shaping offspring’s attention toward emotional faces. For example, 8-month-olds exposed to prenatal or postnatal maternal depression showed greater difficulty in disengaging from fearful faces than infants of mothers with low depression levels (Kataja et al., 2020). Hence, attentional processing of emotional faces is susceptible to the influence of maternal symptoms from early infancy (Porto et al., 2020; Sandre et al., 2022).

The extant studies in youths with depression diagnosis or high-risk (HR) offspring of depressed mothers have used dot-probe, passive viewing, and visual search tasks to measure AB to emotional faces. The dot-probe paradigm (MacLeod et al., 1986) displays an emotional (e.g., sad) and a neutral face side by side, followed by a probe that replaces either the emotional (congruent trial) or neutral stimulus (incongruent trial). Faster responses on the congruent trials indicate an AB toward the emotional faces, whereas a faster response on the incongruent trials indicates AB away from the emotional stimuli. The passive viewing task (e.g., Harrison & Gibb, 2015) presents participants with a set of emotional faces simultaneously (e.g., angry, sad, happy, neutral) over a relatively long (e.g., 20s) free-viewing trial. The key dependent variables are the proportion of times engaged in looking at each emotion type over the total looking time. Lastly, The visual search task (Donnelly et al., 2010; LoBue, 2009; LoBue & Pérez-Edgar, 2014) is designed to evaluate both facilitated detection and difficulty in disengagement from a specific emotional type (Armstrong & Olatunji, 2012). The task presents the participants with a matrix of emotional faces (e.g., 3 × 3 matrix) in each trial. Each matrix contains a single target face with one type of emotional expression (e.g., an angry face) among distracters from another emotion category (e.g., happy faces). Participants are asked to find the target as quickly as they can. For example, faster identification of the angry target among happy distracters indicates facilitated detection of the angry faces, whereas slower identification of the happy target among angry distracters indicates difficulty in disengaging from the angry faces.

Studies that measured participants manual reaction times (RTs) indicated that HR offspring (5–14 years) of depressed mothers displayed AB specifically towards sad faces (e.g., Joormann et al., 2007; Kujawa et al., 2011) in dot-probe tasks, comparable to youth with depression diagnoses (Hankin et al., 2010). Thus, AB towards symptom-related emotional stimuli (e.g., sad faces) is observable in young children, and it is related to risk for depression prior to clinical diagnoses (Lau & Waters, 2017). However, AB can manifest in several components that operate on a time sequence (Cisler & Koster, 2010). RT measures cannot directly capture attention processes with sufficient temporal sensitivity (Yiend, 2010). Moreover, RT measures might not be able to reliably capture the core mechanism underlying AB, given that individual differences in multiple processes may affect RT outcomes (Mogg et al., 2008).

Compared to manual reaction times (RTs), eye tracking (ET) provides a continuous and temporally sensitive measure of eye movements and is well suited to depict the time course and components of AB (Fu & Perez-Edgar, 2019). In a passive viewing task, HR children (8 to 14 years) with depressed mothers showed greater attention maintenance specifically to sad faces, indexed by a greater proportion of time spent fixating on the sad face, compared to children of never-depressed mothers (Owens et al., 2016). Furthermore, HR children’s attention maintenance toward sad faces in an ET dot-probe task increased longitudinally from 8 to 14 years (Gibb et al., 2023). Together, these findings suggest that HR children exhibit increased attention maintenance toward depression-relevant stimuli, an AB pattern comparable with individuals with depression (Mogg & Bradley, 2005). However, existing ET studies that used the visual search task with HR (Platt et al., 2022) and depressed youth (9 to 14 years; Buhl et al., 2021) did not find depression-related group difference in attention disengagement from sad or angry relative to happy distracters.

While ET enables direct assessment of the microstructure of the AB mechanism compared to RT measures, there is a limited understanding of the association between familial risk of depression and attention detection of symptom-relevant emotional stimuli. Behavioral evidence suggests that adulthood depression was associated with a longer time to detection of happy targets (Suslow et al., 2001). Moreover, sensitivity in detecting sad faces may differentiate between a subset of offspring (8 to 14 years) of depressed mothers and those of never-depressed mothers (Burkhouse et al., 2016). However, there is a lack of ET evidence on whether HR children show altered detection of negative and/or positive emotional stimuli. Additionally, the existing RT and ET studies of AB implicated in depression risk have largely been conducted in middle childhood and adolescence. There is a need to study AB patterns in younger HR children before the risk mechanisms are confounded by pubertal changes and the development of compensatory mechanisms.

The emergence of AB to depression-relevant stimuli in HR offspring may be explained by the continuous exposure to maternal depression from early life. Maternal depression provides developmental experiences that fine-tune children’s attention orienting to environmental stimuli related to mothers’ depressive state (e.g., mothers’ sad faces). This may gradually heighten children’s perceptual and neurological sensitivity to detect sad facial expressions and depression-related information in the environment that might not be as salient to others (Smith & Pollak, 2020). The AB may initially be adaptive for the offspring to be affectively attuned to their mothers. However, it can become maladaptive when the AB to depression-relevant stimuli becomes more canalized through development and is applied to a broader range of socioemotional contexts (Burkhouse et al., 2016; Leppanen & Nelson, 2009; Pollak, 2003).

Parent-child association or concordance of AB is another possible mechanism underlying emotion modeling and family aggregation of depression and anxiety (Aktar et al., 2019; Creswell et al., 2010). The genetic and shared environmental factors may contribute to the parent-child concordance of AB (Colich et al., 2017; Ethridge et al., 2022). Parental depressive symptoms and parental AB may sensitize their child to negative stimuli in the environment, inducing AB in the child (Field & Lester, 2010). Supporting evidence in normative samples indicates that both parents and their infants displayed AB towards fearful versus happy faces, measured using ET (Aktar et al., 2021). However, existing studies (Aktar et al., 2019; Mogg et al., 2012; Waters et al., 2015, 2018) that assessed AB using RT measures in children and their mothers with or without a lifetime diagnosis of anxiety and depressive disorders have predominately showed no significant concurrent or prospective associations between parent and child AB (Aktar, 2022 for review). Only one study showed that among HR children, child AB to angry faces was associated with reduced AB to happy faces in mothers when controlling for maternal symptoms (Waters et al., 2015). Given the heterogeneity in maternal diagnoses, we do not yet know the mother-child association of AB as a function of maternal depression diagnostic status. Due to the limitations of manual RT measures of attention, the mother-child association of the specific component of AB also remains unclear.

The present study extends the existing literature by implementing ET to assess AB in mothers with and without a history of MDD and their 4-year-old offspring, an age precedes the onset of clinical-level anxiety and depression symptoms in the offspring. The visual search task was implemented to measure AB in the initial detection and maintenance of the emotional (e.g., sad and angry) target faces in the presence of competing emotional (e.g., happy) distracters. We also explored the association between mothers’ and their offspring’s AB patterns. Based on the extant literature, we hypothesized that HR children of depressed mothers will exhibit AB to sad faces, characterized by faster latency of initial fixation to sad than happy targets and longer dwell time on the sad than happy targets, and LR children with never-depressed mothers will not show AB to sad faces. We also hypothesized that mothers with a history of MDD will show AB to sad faces, comparable to their HR children. Given the mixed findings in the literature about the parent-child association of AB in mothers with depressive symptoms, we did not have a prediction on whether there will be a significant mother-child association of the ET measures, and whether maternal depression history and/or face emotion will moderate the association.

## Method

### Participants

Participants were recruited via electronic media and flyers for a larger study that examined the impact of maternal depression on social cognition in four-year-old offspring. Mothers who responded to the recruitment advertisements were initially screened over the phone to determine potential eligibility and then interviewed using the Structural Clinical Interview for DSM-5 Disorders (SCID-5; First, 2015) in the laboratory. To be eligible for the study, mothers were required to (1) have no history of psychotic symptoms and bipolar disorder and no substance use disorders within the past 6 months; and (2) either meet the criteria for MDD during the target child’s lifetime or no current and history of mood disorders assessed by SCID. Mothers who had met the criteria for MDD were enrolled in the MDD group and those who had no current or lifetime history of MDD were enrolled in the control group. Children were screened using the Wechsler Preschool and Primary Scale of Intelligence– Fourth Edition (WPPSI-IV; Wechsler, 2012) and maternal report on Pervasive Developmental Disorders Screening Test-II (PDDST-II; Siegel, 2021). Eligibility criteria for children were being the biological child of the participating mother, having no developmental disorders (including autism spectrum disorders), and having a full-scale IQ above 70. Only one child per family was enrolled in the study.

Of the 180 mother-child dyads screened, 128 dyads were eligible, and 125 dyads participated in the first assessment wave of the larger study. The present sample was recruited from families who had been enrolled in the initial assessment. A total of 58 children aged four and their mothers participated in the current follow-up study. Reasons for declining continuous participation included a change of address and the COVID-19 pandemic. Children who completed both assessments did not differ from those who only participated in the initial assessment in risk status, *χ*^2^(1) = 0.82, *p* =.37, sex, *χ*^2^(1) = 0.46, *p* =.50, and levels of internalizing symptoms, *t*(93.98)=-1.01, *p* =.31. Of the 58 mother-child dyads, 27 mothers with MDD during their children’s lifetime (*n* = 7 of the mothers also experienced current MDD), 31 mothers with no current or history of MDD, and their offspring participated in the current study. Mothers with a history of MDD reported having experienced on average 4 MDD episodes (*SD* = 3.16; range; 1–10 episodes) since their children were born, with 77.78% (*n* = 21) of the mothers experiencing recurrent MDD episodes since their children were born. Table 1 presents the demographic information of the participants included in the study by group. There were no group differences in child sex and other demographic measures (*p*s > 0.05). All procedures were approved by the Institutional Review Board at the Nationwide Children’s Hospital and The Ohio State University.

**Table 1:** Demographical information of study participants by maternal depression group. There were no group differences in demographic characteristics

	Depressed Mothers (High-Risk Children) *n* = 27	Never-Depressed Mothers (Low-Risk Children) *n* = 31
Offspring Sex (% Males (*n*))	37.04 (10)	58.06 (18)
Offspring Race		
% White (*n*)	74.07 (20)	87.10 (27)
% African American (*n*)	18.52 (5)	12.90 (4)
Mean Offspring Age (SD)	4.05 (0.18)	4.06 (0.16)
Maternal Age	35.31 (4.84)	35.62 (3.43)
Family Income	$70,000 to $79,999	$80,000 to $89,999

### Measures

#### Maternal Depression

During screening, mothers were administered the Mood Episodes module of SCID-5 (First, 2015), a semi-structured clinical interview, to assess their MDD status during the target child’s lifetime. Trained research assistants conducted interviews and recorded maternal MDD status, age of onset, and the number of MDD episodes experienced. 31% of the video-recorded interviews were coded by a second interviewer to assess the interrater reliability of the MDD diagnosis (kappa = 0.92).

#### Child Internalizing Symptoms

Child internalizing symptoms were assessed using the Child Behavior Checklist (CBCL/1.5-5; Achenbach & Rescorla, 2000). Mothers rated their children’s internalizing symptoms using the broad-band internalizing scale consisting of Anxious/Depression, Withdrawn, and Somatic Complaints sub-scales. Each item is rated on a 0–2 scale that asks how well the item describes the child. Raw scores (sum of all relevant items) were used in the current analyses to represent variations in symptom counts, as we planned to examine the effect of internalizing symptom levels on attention patterns to emotional faces.

#### Attention Measures

Attention to emotional faces was assessed using the Visual Search paradigm (Donnelly et al., 2010; LoBue, 2009; LoBue & Pérez-Edgar, 2014). The task stimuli consisted of three sets of 16 photographs each of angry, sad, and happy faces (48 photographs in total). The face stimuli were taken from the NimStim face stimulus set (Tottenham et al., 2009). There were 16 actors with an equal number of male and female faces, as well as White and African American faces. The photographs were arranged in 3 × 3 matrices that contained one target face from one category and eight distracter faces from the paired category to form four task conditions: angry target among happy distracters (AH), happy target among angry distracters (HA), sad target among happy distracters (SH), and happy target among sad distracters (HS). Each of the 16 stimuli served as the target once, and the target appeared in each of the 8 positions (no central position) twice. The 16 distracters appeared an equal number of times across trials. The photographs for each matrix were semi-randomly generated using an in-house Python program. The stimuli were presented on a computer monitor with a built-in eye tracker. The monitor had a diagonal screen size of 23.8 inches with a monitor resolution of 1920 × 1080 pixels. The task presentation was controlled by Tobii Pro Lab software (Tobii Technology AB). A child-size mouse and a regular mouse were used for making manual responses by children and their mothers, respectively.

**Fig. 1:**
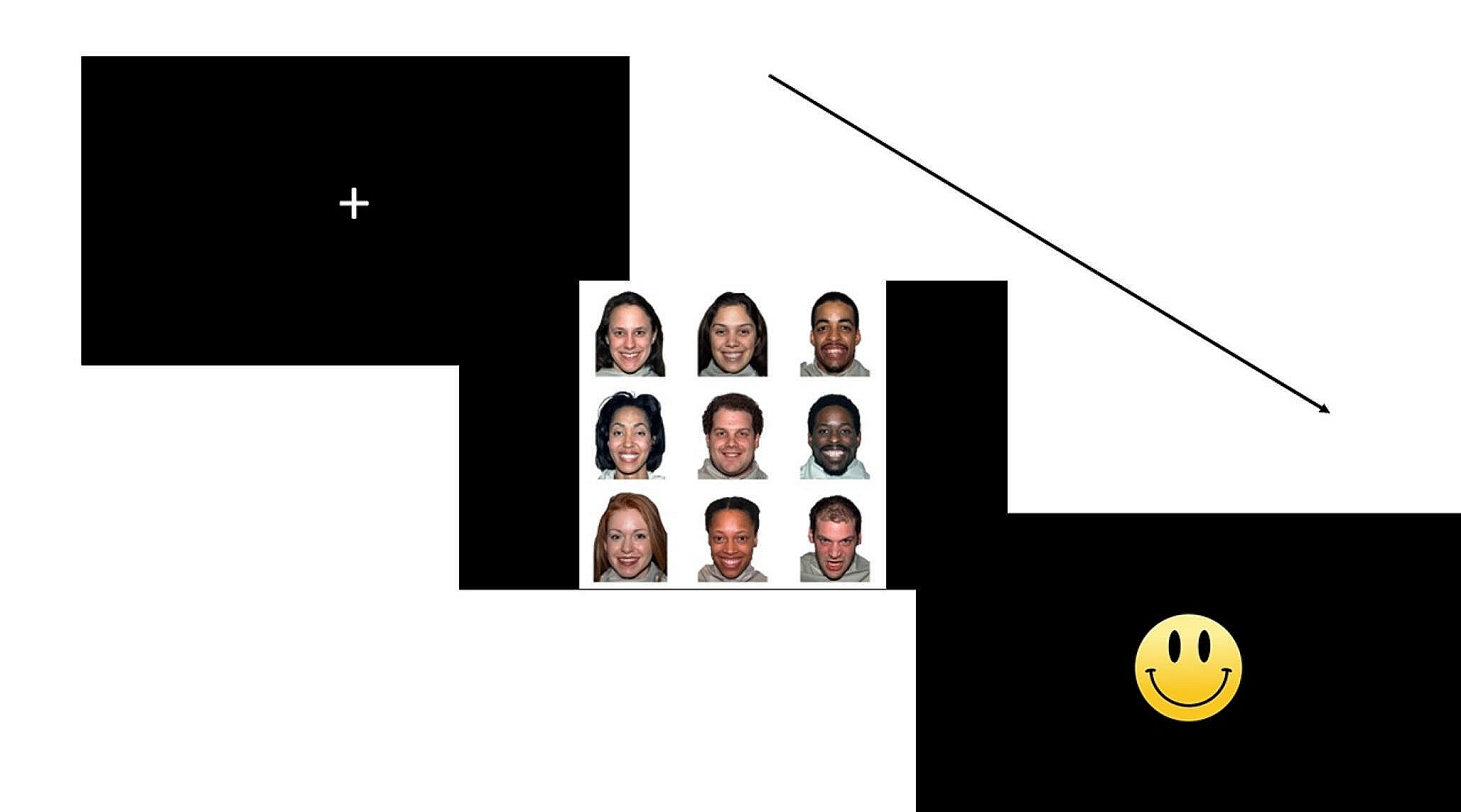
A schematic of the Visual Search Task (Donnelly et al., 2010). The Visual Search task was presented in 4 blocks with 16 trials in each block. Each test trial began with a central fixation cross. The participant was asked to click on the fixation cross. The 3 × 3 matrix was presented upon the mouse click. The participant was instructed to click on the target stimulus as fast as possible using a mouse. Each matrix was presented for 10 s irrespective of a mouse click. A smiley face was displayed in between trials to ensure that the participant’s attention was on the screen. The 4 task conditions were angry target among happy distracters (AH), happy target among angry distracters (HA), sad target among happy distracters (SH), and happy target among sad distracters (HS). There was a short break after each block. Participants were randomly assigned to a block order. Children and their mothers completed identical task procedures

The visual search task was presented in 4 blocks with 16 trials in each block. Figure 1 presents a schematic of the task procedure. First, the experimenter gave instructions about the task by presenting a picture of a target face (e.g., with an angry expression), followed by a distracter face (e.g., with a happy expression). Next, both the target and the distracter were displayed side-by-side. The participant was asked to indicate the target. Lastly, three practice trials followed, each presenting a different 3 × 3 matrix. The test trials only followed if the participant successfully responded to the practice trials. The practice continued until participants were able to correctly identify the three successive targets. Each test trial began with a central fixation cross. The participant was asked to click on the fixation cross. The matrix was presented upon the mouse click. The participant was instructed to click on the target stimulus as fast as possible using a mouse. Each matrix was presented for 10 s irrespective of a mouse click. A smiley face was displayed in between trials to ensure that the participant’s attention was on the screen. A short break followed each block. Participants were randomly assigned to a block order to account for potential practice effects. Children and their mothers completed identical task procedures.

### Procedure

Participants who have completed the initial assessment and agreed to participate in the current study completed the follow-up assessment at our laboratory [blinded]. Mothers completed the questionnaires online or during the initial laboratory visit that took place within 6 months of the follow-up (current) study. Upon arrival at our laboratory, mothers were briefed about the eye tracking assessment procedures and were asked to provide informed consent. Offspring were asked to provide informed assent. Following this, children and their mothers completed the ET visual search task in turn.

### ET Data Acquisition and Processing

The ET data were acquired using Tobii Pro Spectrum (Tobii Technology AB). The screen-based eye tracker has an infrared emitter and camera embedded beneath the monitor that detects and records the reflection of an infrared light source on the cornea relative to the pupil from both eyes. The eye tracker has a sampling rate of 120 Hz, gaze accuracy of 0.3°, and precision of 0.06° (Tobii Technology manufacturer specifications: https://www.tobiipro.com/product-listing/tobii-pro-spectrum/#Specifications). Participants were seated 60 cm from the screen which was placed on a table with adjustable height. Once the experimenter made sure the participant was sitting still and their eye gaze was on the screen, a five-point calibration and a four-point validation procedure were performed using Tobii Pro Lab. Calibration was repeated if the gaze locations from either eye deviated from the locations of the validation points more than 1°. The calibration and validation procedure were repeated before each of the four task blocks.

We exported the gaze data from each participant using Tobii Pro Lab. Fixations were defined using the I-VT fixation classifier (Komogortsev et al., 2010; Salvucci & Goldberg, 2000). The minimum fixation duration was 80 milliseconds with a velocity threshold of 1°/s. An area of interest (AOI) encircling and including the entire location of the individual photograph (9 AOIs in a matrix) was created using Tobii Pro Lab. Data processing was restricted to gaze data within the AOIs. Fixation data to the face AOIs were extracted and processed with in-house Python (Python Software Foundation; Van Rossum & Drake, 2009) and SPSS (Corp, 2019) programs. To reduce potential artifacts resulting from low-quality ET data, we carefully inspected the data and implemented a multistep exclusion procedure. For each participant, we excluded data from the block with unsatisfactory calibration (> 1° deviation). Four LR children (2 males) and two never-depressed mothers did not provide usable ET data. Among 54 children who provided ET data, 51 children provided satisfactory ET data on all trials, with 3 children (2 h children) did not provide usable data for at least one block. Satisfactory ET data were obtained from all trials in 55 mothers, and one mother completed three out of four blocks.

The ET measure of interest is the total dwell time on the target face during the trial. The metrics capture attention maintenance at the targets in the presence of distracters (Armstrong & Olatunji, 2012). Given the task requirement of detecting target faces, we computed the latency of first fixation to the targets. We also recorded manual reaction times (RTs) for detecting the target faces. The ET and RT measures were computed across trials for each of the task conditions (AH, HA, SH, and HS) and each participant. Prior to formal model testing, the internal consistency of the ET and RT measures was estimated using a permutation-based split-half approach with 5000 random splits (*splithalf* R package). Table 2 presented the Spearman-Brown corrected split-half reliability scores (Sears et al., 2019) for the ET and RT measures in children and mothers. The ET dwell time measures had satisfactory reliability in both children and their mothers across stimulus types. However, the ET latency measure had low to medium reliability for most of the stimulus types in children and mothers.

**Table 2:** Spearman-Brown corrected split-half reliability scores for eye-tracking measures from the Visual Search paradigm

	Child					Mother			
	SH	HS	AH	HA		SH	HS	AH	HA
Latency	0.55*	0.20	0.63*		0.53*	0.58*	0.88*	0.23	0.41*
Dwell Time	0.80*	0.74*	0.88*	0.80*		0.97*	0.97*	0.96*	0.97*
Reaction Time	0.75*	0.75*	0.82*		0.67*	0.91*	0.81*	0.87*	0.86*

Note: Dwell Time = total dwell time on the target face during the trial. Latency = latency of first fixation to the target face. SH = sad target happy distracters; HS = happy target sad distracters; AH = angry target happy distracters; HA = happy target and angry distracters. *Significant correlation indicated by 95% confidence interval

### Data Analyses

Exploratory analyses showed that there were no group differences in child sex, *p* =.11, and internalizing levels, *p* =.09. However, child sex and internalizing symptoms were still included as covariates in all models with the child data as the outcome, given their effects on attention patterns in children (Bar-Haim et al., 2007; Kujawa et al., 2011). The first set of main analyses examined the effects of maternal MDD history or group (LR versus HR) and target stimulus type on children’s latency of initial target fixation. Four separate linear mixed effects (LME) models (Pinheiro et al., 2022) were fitted using R (R Core Team, 2021) to examine the between-persons effect of maternal MDD history, within-person effect of stimulus type (sad versus happy, or angry versus happy), and their interaction effect, on children’s latency of first fixation and dwell time on the targets, respectively. Next, four separate LME models were fitted to test the effect of maternal MDD history, stimulus type (sad versus happy, or angry versus happy), and their interaction effect, on the mother’s latency of first fixation and dwell time on the targets, respectively. Table 3 presents descriptive statistics for the continuous variables entered in the main analyses.

The results of the additional analyses are presented in Supplementary Information (SI). First, LME models were conducted to examine whether maternal MDD history and stimulus type moderate the association between mothers’ and children’s latency of initial fixation and mean dwell time on the target faces. The analyses are exploratory as our sample size does not permit adequate testing of three-way interactions effects of group, stimulus type and mothers’ ET measure. Second, we repeated the above main and additional analyses by using maternal BDI scores to index maternal current depressive symptoms. Third, additional analyses also examined the impacts of maternal depression (MDD history or BDI scores), stimulus type, and their interaction effects on children’s RT measures and mothers’ RT measures. Lastly, among HR children, we examined whether the number of MDD episodes that mothers experienced since their children were born moderated the effect of stimulus type on children’s ET measures.

**Table 3:** Mean (standard deviation) for the maternal reports and eye-tracking measures

	Depressed Mothers (High-Risk Children)	Never-Depressed Mothers (Low-Risk Children)
**Maternal Reports**		
CBCL internalizing scores	6.78(5.63)	4.32(5.17)
**Child Eye-tracking Measures (milliseconds)**		
Mean latency sad target	2934.96(928.57)	3140.92(679.00)
Mean latency happy target in HS trials	3841.82(725.93)	3437.09(681.72)
Mean latency angry target	3199.35(791.71)	3295.61(1179.23)
Mean latency happy target in HA trials	3659.66(863.26)	3873.45(1163.26)
Mean dwell time sad target	2153.57(692.54)	1843.71(694.54)
Mean dwell time happy target in HS trials	1454.65(556.11)	1536.59(539.23)
Mean dwell time angry target	2191.27(995.03)	2040.43(874.24)
Mean dwell time happy target in HA trials	1385.62(599.43)	1386.08(597.56)
**Maternal Eye-tracking Measures (milliseconds)**		
Mean latency sad target	1379.74(274.59)	1248.55(244.79)
Mean latency happy target in HS trials	1454.98(331.87)	1347.90(343.70)
Mean latency angry target	1208.64(209.16)	1218.32(241.30)
Mean latency happy target in HA trials	1467.89(276.42)	1454.35(269.07)
Mean dwell time sad target	4249.28(2225.61)	3516.94(1797.87)
Mean dwell time happy target in HS trials	3393.20(1987.04)	3477.21(1819.75)
Mean dwell time angry target	4173.01(2077.54)	3549.37(1687.15)
Mean dwell time happy target in HA trials	3982.688(2005.64)	3573.14(1936.54)

*Notes*: HS = happy-sad; HA = happy-angry

## Results

### The Effects of Maternal MDD History and Stimulus type on Children’s Latency of Initial Fixation on the Targets

**Fig. 2:**
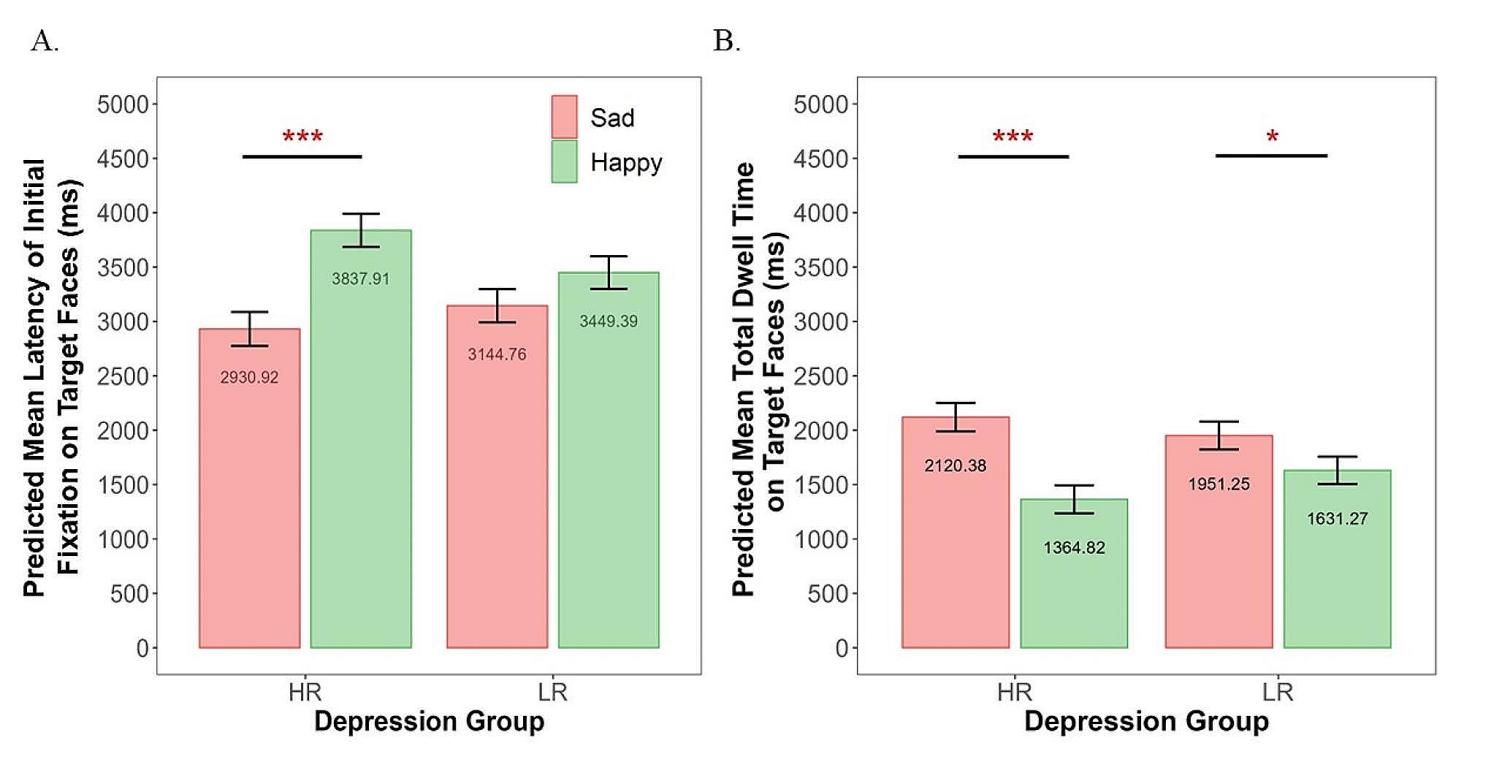
Group-by-stimulus interaction effect on the latency and dwell time measures on sad and happy target faces in low-risk (LR; with never-depressed mothers) and high-risk (HR; with depressed mothers) children. **A**. The significant interaction effect on the mean latency of initial fixations towards the targets. **B**. The significant interaction effect on the mean total dwell time on the targets. *Note*: *p <.05, ***p <.001

We examined the effect of group (HR versus LR), stimulus type, and their interaction effect on children’s latency of initial fixation to the target faces, separately for sad-happy and angry-happy conditions. The parameter estimates of the models and the effect sizes (*R*^2^) for the fixed effects are presented in Table 4. For the sad-happy condition (Model 1a), there was no effect of sex, *p* =.65, internalizing levels, *p* =.64, or group difference in the initial detection latency across sad and happy target faces, *p* =.61. There was a main effect of stimulus type, *F*(1,43) *=* 19.61, *p* <.001, indicating that children were faster in detecting sad than happy targets across both groups, *B*=-302.90, *SE* = 70.64, *t*=-4.29, *p* <.001. There was also a significant group-by-stimulus interaction effect, *F*(1,43) = 4.81, *p* =.03. Figure 2A presents the interaction effect. The interaction effect suggests that the difference in fixation latency between sad and happy targets was greater in the HR than LR group, *B*=-150.59, *SE* = 70.58, *t*=-2.13, *p* =.04. Specifically, the HR children were faster in initially fixating on the sad than happy targets, *B*=- 453.49, *SE* = 100.85, *t*= -4.50, *p* <.001, whereas their LR peers did not show different latency in fixating on sad versus happy targets, *p* =.13. There was no group difference in latency to fixate on the sad targets, *p* =.36, or Happy targets, *p* =.09. For the angry-happy condition (Model 1b), there was only a significant effect of stimulus type, *F*(1,43) *=* 11.04, *p* =.002, indicating faster detection for angry than happy targets across all children, *B*=-268.28, *SE* = 83.39, *t*=-3.22, *p* =.003. There was no significant effect of group, *p* =.51, or group-by-stimulus interaction effect, *p* =.71.

### The Effects of Maternal MDD History and Stimulus type on Children’s mean Dwell time on the Targets

For the sad-happy condition (Model 2a), there was no effect of sex, *p* =.25, internalizing symptoms, *p* =.26, or maternal MDD history on the mean dwell time on the target faces when collapsed across emotion type, *p* =.76. However, there was a significant stimulus type effect, *F*(1,43) = 32.26, *p* <.001, such that children across both groups detected the sad targets faster than happy targets, *B* = 268.89, *SE* = 48.89, *t* = 5.50, *p* <.001. There was a significant group-by-stimulus interaction effect, *F*(1,43) = 5.30, *p* =.03, indicating that the difference in dwell time on sad versus happy targets was significantly greater in the HR than LR group (Fig. 2B), *B* = 108.90, *SE* = 48.86, *t* = 2.23, *p* =.03. The mean dwell time was longer for sad than happy targets in the HR, *B* = 377.78, *SE* = 69.83, *t* = 5.41, *p* <.001, and LR children, *B* = 159.99, *SE* = 68.40, *t* = 2.34, *p* =.02. There were no group differences in dwell time on the sad or happy targets, *p*s > 0.16. For the angry-happy condition (Model 2b), there was a significant effect of stimulus type, *F*(1,44) *=* 37.87, *p* <.001, such that the dwell time was longer on angry than happy targets across both groups, *B* = 362.24, *SE* = 60.77, *t* = 5.96, *p* <.001. There was no significant effect of group, *p* =.75, or group-by-stimulus interaction effect, *p* =.49.

### The Effects of Maternal MDD History and Stimulus type on Mothers’ Latency of Initial Fixation on the Targets

For the sad-happy condition (Model 3a), no significant effect of group, *p* =.09, or stimulus type, *p* =.07 was found for the latency measure. There was also no group difference in the latency of initial fixation towards the sad versus happy targets, *p* =.60. For the angry-happy condition (Model 3b), there was a significant effect of stimulus type, *F*(1,50) = 55.73, *p* <.001, indicating that mothers were faster in detecting angry than happy targets across both groups, *B*=-123.61, *SE* = 16.88, *t*=-7.32, *p* <.001. There was no significant effect of group, *p* =.97, or group-by-stimulus interaction effect, *p* =.72.

### The Effects of Maternal MDD History and Stimulus type on Mothers’ Dwell time on the Targets

**Fig. 3:**
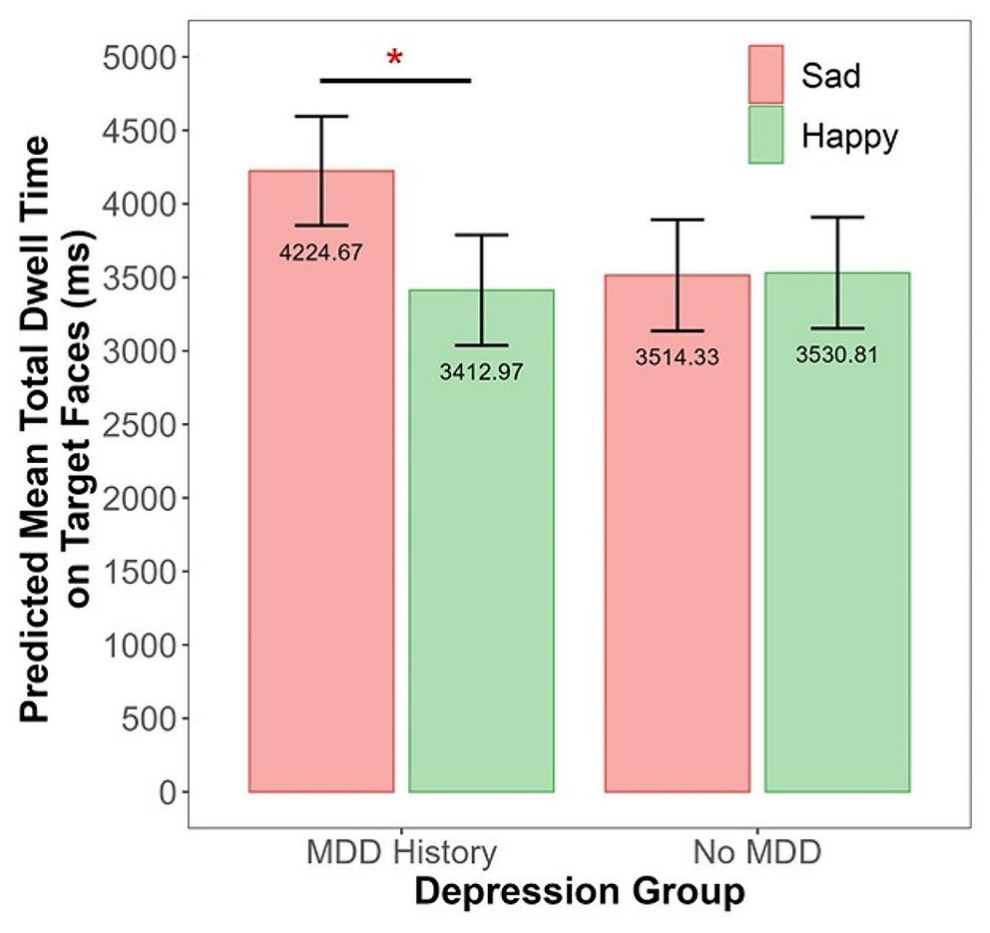
Group-by-stimulus interaction effect on the mean total dwell time on the sad and happy target faces in mothers with and without a history of major depressive disorder (MDD). *Note*: *p <.05

For the sad-happy condition (Model 4a), there was no significant effect of group, *p* =.54, stimulus type, *p* =.08, or group-by-stimulus interaction, *p* =.07. We probed the interaction effect, given our hypothesis that maternal depression is associated with AB to sad faces. Indeed, mothers with a history of MDD exhibited longer dwell time on the sad than happy targets, *B* = 405.85, *SE* = 161.96, *t* = 2.51, *p* =.02, whereas the dwell time did not differ between emotion types in mothers with no MDD history, *p* =.96 (Fig. 3). For the angry-happy condition (Model 4b), there was no significant effect of group, *p* =.30, stimulus type, *p* =.67, or group-by-stimulus interaction effect, *p* =.53, on mothers’ mean total dwell time on the targets.

**Table 4:** Results from the linear mixed-effects models

Parameters	Est.	95% CI	*R* ^2^
**Model 1a: outcome: mean latency of initial target fixation in children**			
**Fixed effects**			0.18
Intercept	**3298.23**	(3051.80, 3544.66)	
Sex	78.24	(-264.49, 420.95)	
CBCL internalizing score	-7.72	(-40.34, 24.91)	
Group	43.67	(-129.04, 216.38)	
Stimulus (sad vs. happy target)	**-302.90**	(-440.83, -164.97)	
Group×Stimulus	**-150.59**	(-288.41, -12.76)	
**Random effects**			
SD Intercept	324.60	(149.65, 704.10)	
Residual	665.07	(541.73, 816.50)	
**Model 1b outcome: mean latency of initial target fixation in children**			
**Fixed effects**			0.09
Intercept	**3504.18**	(3051.99, 3756.73)	
Sex	196.07	(-301.20, 693.33)	
CBCL internalizing score	-22.15	(-67.25, 22.94)	
Group	-82.67	(-334.21, 168.88)	
Stimulus (angry vs. happy target)	**-268.28**	(-431.11, -105.45)	
Group×Stimulus	30.55	(-132.23, 193.33)	
**Random effects**			
SD Intercept	600.00	(396.89, 907.05)	
Residual	780.82	(635.91, 958.76)	
**Model 2a outcome: mean total dwell time on the targets in children**			
**Fixed effects**			0.21
Intercept	**1646.81**	(1419.31, 1874.30)	
Sex	230.13	(-86.01, 546.27)	
CBCL internalizing score	17.20	(-13.07, 47.48)	
Group	-24.33	(-183.88, 135.21)	
Stimulus (sad vs. happy target)	**268.89**	(173.42, 364.35)	
Group×Stimulus	**108.90**	(13.49, 204.30)	
**Random effects**			
SD Intercept	424.14	(294.75, 610.33)	
Residual	456.47	(368.13, 566.01)	
**Model 2b outcome: mean total dwell time on the targets in children**			
**Fixed effects**			0.23
Intercept	**1866.13**	(1596.56, 2135.69)	
Sex	-225.03	(-603.16, 153.09)	
CBCL internalizing score	29.87	(-4.60, 64.35)	
Group	30.92	(-160.74, 222.58)	
Stimulus (angry vs. happy target)	**362.24**	(243.61, 480.87)	
Group×Stimulus	40.79	(-77.81, 159.39)	
**Random effects**			
SD Intercept	475.47	(325.87, 693.73)	
Residual	573.18	(467.59, 702.63)	
**Model 3a outcome: mean latency of initial target fixation in mothers**			
**Fixed effects**			0.06
Intercept	**1360.48**	(1288.98, 1431.98)	
Group	62.25	(-9.25, 133.75)	
Stimulus (sad vs. happy target)	-38.29	(-80.31, 3.73)	
Group×Stimulus	11.39	(-30.63, 53.41)	
**Random effects**			
SD Intercept	210.01	(152.73, 288.76)	
Residual	210.49	(172.27, 257.19)	
**Model 3b outcome: mean latency of initial target fixation in mothers**			
**Fixed effects**			0.21
Intercept	**1337.08**	(1277.97, 1396.20)	
Group	1.18	(-57.93, 60.29)	
Stimulus (angry vs. happy target)	-123.61	(-156.87, -90.35)	
Group×Stimulus	-6.02	(-39.27, 27.24)	
**Random effects**			
SD Intercept	177.22	(132.56, 236.92)	
Residual	169.14	(139.67, 204.82)	
**Model 4a outcome: mean total dwell time on the targets in mothers**			
**Fixed effects**			0.03
Intercept	**3670.70**	(3187.38, 4154.02)	
Group	148.13	(-335.19, 631.44)	
Stimulus (sad vs. happy target)	198.81	(-27.17, 424.78)	
Group×Stimulus	207.04	(-18.94, 433.02)	
**Random effects**			
SD Intercept	1549.23	(1205.89, 1990.31)	
Residual	1149.25	(947.72, 1393.64)	
**Model 4b outcome: mean total dwell time on the targets in mothers**			
**Fixed effects**			0.02
Intercept	**3822.40**	(3331.70, 4313.09)	
Group	255.45	(-235.24, 746.15)	
Stimulus (angry vs. happy target)	38.80	(-142.30, 219.89)	
Group×Stimulus	56.37	(-124.73, 237.46)	
**Random effects**			
SD Intercept	1653.62	(1324.66, 2064.27)	
Residual	919.90	(759.35, 1114.38)	

Note: Est.=beta values; CI = Confidence interval; Estimates are bolded if their 95% CI does not contain zero, suggesting a significant effect; CBCL = Child Behavior Checklist; *p* values are reported in the text

## Discussion

The present study aimed to examine (1) whether children with familial risk of depression (HR) displayed different AB patterns relative to their LR peers, and (2) whether mothers with a history of MDD exhibit comparable AB patterns as their children. Additionally, we directly examined the mother-child association in the attention measures. We implemented an ET visual search task (Armstrong & Olatunji, 2012; Donnelly et al., 2010; LoBue, 2009; LoBue & Pérez-Edgar, 2014) to examine AB in children at 4 years of age, a developmental period that precedes the onset of clinically significant levels of psychopathology but is marked by increased incidence of socioemotional problems (Avenevoli et al., 2008; Egger & Angold, 2006).

Our primary findings revealed both normative AB patterns across all children as well as AB patterns specific to children at familial risk for MDD. In the visual search task, AB is manifested as faster detection (i.e., shorter latency) and/or longer maintenance (i.e., greater dwell time) of the negative emotional targets relative to happy targets. We found that children across both groups displayed AB to angry targets relative to happy targets indexed by both measures. Children from both groups exhibited longer attention maintenance on the sad relative to happy targets after the targets were located. However, there was a significant interaction effect, reflected by the findings that the difference in the predicted values between mean dwell time on the sad versus happy targets was greater in the HR than LR group. Furthermore, the HR children were faster in detecting the sad than happy targets, whereas the LR children did not show such pattern of sad bias. We did not find significant group-by-stimulus interaction effect on mothers’ ET measures. Although there was an indication that mothers with a history of MDD had longer attention maintenance to sad relative to happy targets, the non-significant interaction effect suggests that AB to sad faces in mothers with an MDD history is not significantly greater than in never-depressed mothers. Our supplementary results (see SI) revealed that there were no mother-child associations in AB to angry or sad targets. Maternal MDD history did not moderate the mother-child associations in AB patterns.

Our findings indicate potential normative AB towards threat-related (i.e., angry faces) in early development. Both LR and HR in the current study showed longer attention maintenance to sad relative to happy targets. Children across both groups also displayed angry bias. This is consistent with findings that HR and LR children did not differ in AB patterns (Gibb et al., 2009; Waters et al., 2015). AB to threat-related faces might be normative and early emerging in development (e.g., Leppanen & Nelson, 2009; LoBue & DeLoache, 2010; LoBue & Pérez-Edgar, 2014; Nakagawa & Sukigara, 2019). However, maladaptive AB may stem from the normative AB patterns for a subgroup of children who are at risk for internalizing problems (Morales et al., 2016).

Indeed, children with familial risk of MDD also displayed a distinct pattern of sad bias compared to their LR peers. Cognitive theories based on adult research suggest that depression is associated with increased attention allocation to dysphoric or sad stimuli and reduced attention allocation to anhedonic or rewarding stimuli (Armstrong & Olatunji, 2012; Suslow et al., 2020). Offspring of mothers with an MDD history displayed facilitated detection to the sad than happy targets, whereas this sad bias was not found in their LR peers. The findings were consistent with the existing evidence that risk for depression in childhood is linked to heightened sensitivity in detecting sad faces (Burkhouse et al., 2016) or potentially blunted sensitivity in detecting socially rewarding information as shown in adults with depression (Suslow et al., 2001). Moreover, HR children exhibited greater attention maintenance to sad than happy targets, and this sad bias pattern was greater in HR than LR children. However, different from existing evidence (Owens et al., 2016), HR children did not show longer attention maintenance specifically on the sad targets compared to LR children. One possibility is that LR healthy controls also show sad AB that takes place in the later stage of information processing in tasks that require goal-directed attention orienting based on task demands (Buhl et al., 2021; Platt et al., 2022; Sylvester et al., 2016). Thus, the difference in sad bias between LR and HR children might be diminished in these tasks compared to passive viewing tasks (Owens et al., 2016).

The current findings do not support a significant mother-child association of AB. Mothers with an MDD history exhibited longer attention maintenance to sad than happy targets. Hence, there is a similarity of the sad AB pattern towards symptom-relevant stimuli between mothers with MDD history and their HR children. However, the sad bias in mothers with an MDD history was not significantly greater than in those never-depressed mothers. Furthermore, we did not find a positive relation between fixation latency and dwell time on the target faces. Additionally, our findings do not indicate that the mother-child association of AB differs depending on maternal MDD history. Our finding is consistent with Aktar et al. (2021) who did not find a significant moderating effect of parental symptoms on the parent-infant association in AB to emotional faces. Hence, we cannot conclude that offspring who display AB to sad faces modeled their attention patterns from their mothers. It is possible that additional mechanisms contributed to the emergence of AB in the HR offspring.

Future studies are needed to examine parental affective expressions and parenting behavior in mothers with MDD history. Depressed mothers tended to provide less sensitive parenting (Murray et al., 2010) and display less positive and more neutral or negative emotions (Aktar et al., 2017). The early-life experience of symptom-relevant stimulus exemplars and caregiving behaviors may serve to canalize the offspring’s attention to the types of stimuli that they are most likely to encounter (Leppanen & Nelson, 2009; Pollak, 2003). Environmental exposure to mothers’ negative emotional expressions and parenting behavior may gradually heighten children’s perceptual and neurological sensitivity to detect sad faces and depression-related information in the environment, thus developing “experience-specific information-processing biases” over time (Burkhouse et al., 2016; Leppanen & Nelson, 2009; Pollak, 2003). Additionally, the neural systems underlying attention to facial emotion are particularly malleable during the first several years of life (Blair & Raver, 2012; Fox et al., 2010). A future direction is to examine whether parental affect and parenting behavior influence the link between maternal MDD and offspring’s AB patterns.

Limitations need to be considered when interpreting current findings. First, our analyses using multiple indices of AB (ET dwell time, ET latency, and manual RTs) in a relatively small sample size were exploratory. Our HR group contained a larger proportion of females than males, although there was no significant group difference in sex. Future studies with larger sample size can benefit from testing whether there are sex differences in the effect of maternal depression on the offspring’s AB patterns (e.g., Kujawa et al., 2011). Moreover, there are between-subjects variations in the time interval between data collections of questionnaires and ET measures. This methodological issue complicates interpretations about the impacts of child internalizing symptoms (measured by CBCL) or maternal depressive levels (measured by Beck Depression Inventory described in SI) on ET measures of AB patterns.

Second, the ET measure of latency to initial target fixation had unsatisfactory reliability. Future studies can benefit from implementing multiple ET task paradigms to capture AB operating at different stages of information processing (Fu & Perez-Edgar, 2019). The rich set of ET measures obtained from multiple tasks can be used to characterize AB profiles associated with familial risk for depression (variable-centered analysis) or characterize sub-groups of HR children (person-centered analysis) (Vallorani et al., 2021).

Third, the present study did not examine parenting styles or parent-child interaction dynamics directly. Evidence supports the association between child AB and negative parenting (Gulley et al., 2014) or parent-child conflicts (Connell et al., 2013). Future studies are needed to characterize the environmental factors that mediate the impact of maternal depression on the emergence of AB in the offspring.

Fourth, the current study cannot speak to the impacts of timing and chronicity of exposure to maternal depression on the development of AB. Postnatal exposure to maternal depression may disrupt the development of neural networks underlying attention control and emotion regulation that are plastic from the prenatal period to childhood (Loman & Gunnar, 2010). Further evidence is needed to assess whether offspring’s AB patterns and mother-child association of AB may vary with the age of maternal depression exposure.

Fifth, we do not provide longitudinal evidence that examines whether AB to sad faces predicts the emergence of socioemotional problems and psychopathology in children. We also did not examine the directionality of parent-child relations in AB or depression levels. While Waters et al. (2018) did not find a cross-sectional relation between mothers’ and children’s AB, they revealed children’s AB at the first assessment was associated with their mothers’ AB at the second assessment, indicating a child-to-parent transmission of AB. Longitudinal evidence has also pointed to the bidirectional links between parent-adolescent depressive symptoms (Johnco et al., 2021). Future longitudinal studies are needed to examine the complex bidirectional influences between AB and psychopathology in parent-child dyads (Aktar, 2022; Pérez-Edgar et al., 2021).

Lastly, future investigations are needed to investigate separate contributions of mothers’ and fathers’ depression on children’s AB. It is possible that depression and anxiety in mothers may have a distinct impact on the offspring’s AB patterns (Aktar et al., 2016, 2019). Studies that evaluate the impact of depression in both parents may be able to better account for the variability in the offspring’s AB.

In conclusion, the present study examined the impact of maternal MDD history on AB towards emotional faces in four-year-olds. We also examined AB patterns in mothers to investigate whether there is a concordance in AB patterns between mothers with a history of MDD and their offspring. We found both normative AB patterns across all children as well as AB patterns specifically associated with familial risk of depression. AB to threat-related (e.g., angry) faces might be normative and emerge early in development. Maternal MDD history was associated with children’s facilitated detection of sad relative to happy faces. The facilitated detection of sad faces was not found in children with never-depressed mothers. Children across both groups exhibited greater attention maintenance to sad than happy faces, although the sad bias indexed by dwell time was greater in children of depressed mothers than those with never-depressed mothers. The present study did not find mother-child association in AB patterns. Future research needs to directly examine the possibility that maternal MDD may also have created a psychosocial environment that gradually shaped their children’s attention towards symptom-congruent socioemotional information.

## Electronic Supplementary Material

Below is the link to the electronic supplementary material.

Supplementary Material 1
