# Supplementary material for: Young Children of Mothers with a History of Depression Show Attention Bias to Sad Faces: An Eye-tracking Study
Source: Res Child Adolesc Psychopathol. 2024 May 7;52(9):1469–83. doi: 10.1007/s10802-024-01205-w (PMC11420269; doi:10.1007/s10802-024-01205-w)
Supplement: Supplementary file 1 — Supplementary Material 1 [file 10802_2024_1205_MOESM1_ESM.docx]

Supplementary Information

**Additional maternal depression measure**

Mothers who participated in the study completed the Beck Depression Inventory (BDI; Beck et al., 1996) that measured the presence and severity of depression. The BDI-II is a 21-item inventory. Each item is rated on a 0-3 scale, yielding summary scores that range between 0 and 63. Mothers with current or MDD diagnosis during the child’s lifetime (*M*=20.48, *SD*=11.80) had higher BDI-II scores than non-depressed mothers (*M*=5.54, *SD*=5.05), *t*(34.2)=6.13, *p*<.001.

**Supplementary results**

The Supplementary Information (SI) presents results from additional linear mixed effects (LME) models examined. The parameter estimates of all models and the effect sizes (*R*^2^) for the fixed effects are presented in SI Table 1. In part one, LME models were conducted to test whether maternal depression (MDD history or current depression levels) and target stimulus type (sad versus happy, or angry versus happy) moderated the association between mothers’ and children’s eye tracking (ET) measures, including the latency of initial fixation and mean dwell time on the target faces. In part two, we examined the effects of maternal current depressive levels and stimulus type on children’s and their mothers’ ET measures. In part three, results are presented for the effects of maternal depression (MDD history or current depressive levels) and stimulus type on children’s and their mothers’ manual reaction time (RT) measures. SI Table 2 provides the mean and standard deviations of the RT scores from the children and their mothers. In part four, we investigated whether the number of MDD episodes that the mothers experienced since their HR children were born moderated the effect of stimulus type on children’s ET measures. Child sex and internalizing symptoms were included as covariates in all models with the child data as the dependent variable.

**1.1. The effects of maternal MDD history, stimulus type, and maternal ET measure on children’s latency of initial fixation on the targets**

We explored whether the mother-child association in the latency of the first fixation to the target faces differs by maternal MDD history (or group) and stimulus type, separately for sad-happy and angry-happy condition. For the sad-happy condition, mothers’ latency of initial fixation to the target faces were not related to the children’s latency measure, *p*=.76. Maternal MDD history, *p*=.12, or stimulus type, *p*=.11, did not moderate the mother-child association of the latency measure. There was also no significant three-way interaction effect between group, stimulus type, and maternal latency measure on the children’s initial fixation latency on the targets, *p*=.26. For the angry-happy condition, the effect of mothers’ latency of initial fixation to the targets was significant, *F*(1,37)*=*5.04, *p*=.03, such that there was a negative mother-child association in the latency of target fixation across angry and happy targets, *B*=-0.99, *SE*=0.47, *t*=-2.12, *p*=.04. No two-way or three-way interaction effects were significant, *p*s>.34.

**1.2. The effects of maternal MDD history, stimulus type, and maternal ET measure on children’s mean dwell time on the targets**

For the sad-happy condition, there was no mother-child association in dwell time on the targets across groups and stimulus types, *p*=.11. However, maternal MDD history, *p*=.37, or stimulus type, *p*=.89, or a combination of both factors, did not moderate the mother-child relation in the dwell time measure, *p*=.56. Similarly, for the angry-happy condition, mothers’ dwell times on the targets were not related to their children’s dwell measure across group and stimulus type, *p*=.98. Maternal MDD history, *p*=.97, or stimulus type, *p*=.38, did not moderate the mother-child association of the target dwell time. There was also no significant three-way interaction effect, *p*=.39.

**2.1. The effects of maternal depression level and stimulus type on children’s latency of initial fixation on the targets**

We examined the effect of maternal depression level (i.e., BDI score), stimulus type, and their interaction effect on children’s latency of initial fixation to the target faces, separately for sad-happy and angry-happy conditions. For the sad-happy condition, there was no significant effect of maternal current depression level on the latency of first fixation on the target faces in children, *p*=.23. There was a significant effect of stimulus type, *F*(1,43)=18.70, *p*<.001. Specifically, children showed faster initial fixation to the sad than happy targets across maternal depression levels, *B*=-306.02, *SE*=73.09, *t*=-4.19, *p*<.001. The interaction effect of maternal depression level and stimulus type was not significant, *p*=.24. For the angry-happy condition, there was also a significant effect of stimulus type, *F*(1,43)=11.06, *p*=.002, such that children showed faster detection of the angry than happy targets across maternal depression levels, *B*=-264.17, *SE*=82.03, *t*=-3.22, *p*=.002. There were no other significant main effects, *p*>.25, and the depression-by-stimulus interaction effect was not significant, *p*=.22.

**2.2. The effects of maternal depression level and stimulus type on children’s mean dwell time on the targets**

For the sad-happy condition, there was a significant effect of maternal depression level, *F*(1,47)=12.47, *p*=.001, such that greater maternal depression level was related to shorter dwell time on the target faces in children, *B*=-25.52, *SE*=7.47, *t*=-3.42, *p*=.001. There was also a significant effect of stimulus type, *F*(1,43)=35.11, *p*<.001, such that children showed longer dwell time on sad than happy targets across maternal depression levels, *B*=275.49, *SE*=48.02, *t*=5.74, *p*<.001. Importantly, there was a significant interaction effect between maternal depression level and stimulus type, *F*(1,43)=5.19, *p*=.03, indicating that greater maternal depression level was associated with greater difference in children’s dwell time on sad versus happy targes, *B*=9.72, *SE*=4.41, *t*=2.20, *p*=.03. SI Figure 1 shows that children with mothers reporting high depression levels displayed greater dwell time on the sad than happy targets, *B*=388.25, *SE*=73.37, *t*=5.29, *p*<.001. Children with less depressed mothers also exhibited greater dwell time on the sad than happy targets, *B*=162.74, *SE*=66.77, *t*=2.44, *p*=.02. Greater maternal depression level was associated with shorter dwell time on happy targets in children, *B*=-35.24, *SE*=8.67, *t*=-4.06, *p*<.001, whereas the effect of maternal depression on dwell time on the sad targets were not significant, *p*=.07. For the angry-happy condition, there was a significant effect of stimulus type, *F*(1,44)=37.03, *p*<.001, such that children had longer dwell time on angry than happy targets, *B*=239.74, *SE*=60.81, *t*=5.89, *p*<.001. There was no effect of maternal depression level, *p*=.17, and stimulus type did not moderate the effect of maternal depression on target dwell time, *p*=.57.


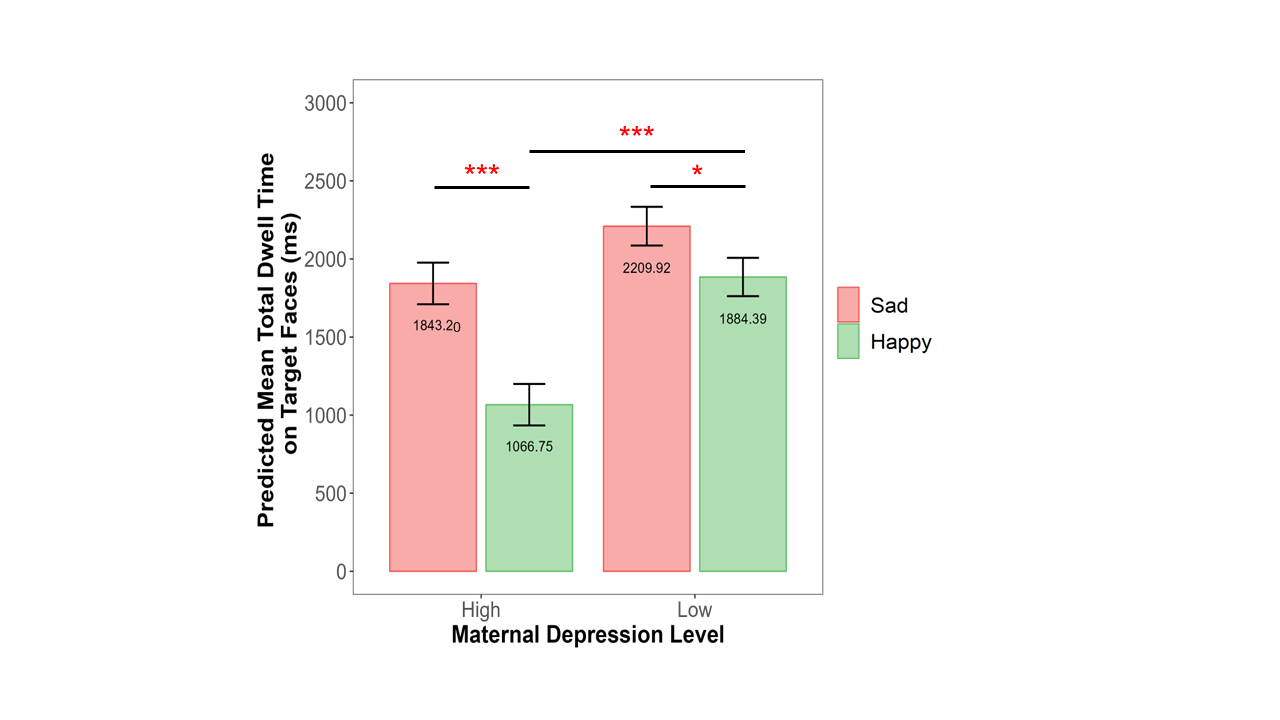


**SI Figure 1:** The interaction effect of maternal depression level, indexed by the scores on the Beck Depression Inventory (BDI) and stimulus type (sad versus happy target faces) on children’s dwell time on target faces.

*Note*: **p*<.05, ****p*<.001

**2.3. The effects of maternal depression level and stimulus type on mothers’ latency of initial fixation on the targets**

For the sad-happy condition, there was no significant effect of maternal current depression level on the latency of first fixation on the target faces in mothers, *p*=.65. Although trend level effect of stimulus type was found for the latency measure, *p*=.08, there was no significant interaction effect of maternal depression level and stimulus type, *p*=.86. For the angry-happy condition, there was a significant effect of stimulus type, *F*(1,50)=55.88, *p*<.001, such that mothers had faster latency of first fixation on the angry than happy targets, *B*=-123.82, *SE*=16.89, *t*=-7.33, *p*<.001. There was no effect of maternal depression, *p*=.77, or depression-by-stimulus interaction effect, *p*=.85.

**2.4. The effects of maternal depression level and stimulus type on mothers’ dwell time on the targets**

Maternal depression level did not significantly impact mothers’ mean total dwell time across the sad and happy targets, *p*=.11. There was a trend level effect of stimulus type, *p*=.08. The depression-by-stimulus-type effect was not significant on the dwell time measure in mothers, *p*=.13. For the angry-happy condition, there was a trend level effect of maternal depression level, *p*=.08. The main effect of stimulus type, *p*=.66, and the depression-stimulus interaction effect were not significant, *p*=.69.

**3.1. The effects of maternal depression level, stimulus type, and maternal ET measure on children’s latency of initial fixation on the targets**

We examined whether mothers’ current depression levels and stimulus type (sad versus happy, or angry versus happy) moderated the mother-child association in the latency of initial target fixations. For the sad-happy condition, there was no mother-child association in the latency measure, *p*=.87. However, maternal depression level moderated the mother-child association, *F*(1,36)=6.58, *p*=.01. Despite the non-significant simple slopes, less depressed mothers (-1SD) and their children displayed positive association of their initial fixation latency of the targets, *B*=0.82, *SE*=0.44, *t*=1.85, *p*=.07, whereas there was a negative association of the latency measure among more depressed mothers and their offspring, *B*=-0.72, *SE*=0.42, *t*=-1.71, *p*=.10. Stimulus type, *p*=.15, or the combination of maternal depression level and stimulus type, *p*=.51, did not moderate the mother-child association of the latency measure. For the angry-happy condition, there was a mother-child association in the latency of initial fixations on the target faces, *F*(1,37)=5.37, *p*=.03, such that faster detection of the target faces in children was related to longer latency of initial target face fixations in mothers, *B*=-1.04, *SE*=0.47, *t*=-2.19, *p*=.03. Maternal depression level, *p*=.89, stimulus type, *p*=.74, and the combination of both factors did not moderate the mother-child association in the latency measure, *p*=.51.

**3.2.** **The effects of maternal depression level, stimulus type, and maternal ET measure on children’s dwell time on the targets**

For the sad-happy condition, there was a main effect of maternal depression level, *F*(1,46)=11.29, *p*=.002, and there was a marginally significant association between mothers and their children’s dwell time on target faces, *p*=.07. Maternal depression level, *p*=.19, or stimulus type, *p*=.99, or a combination of both factors, did not moderate the mother-child association in the dwell time measure, *p* =.91. Similarly, for the angry-happy condition, there was no significant main effect of maternal depression level, *p* =.10, and maternal dwell time on the target, *p* =.97, and the two-way and three-way interaction effects were not significant, *p*s>.29.

**4.1. The effects of maternal MDD history and stimulus type on children’s reaction time (RT) measure**

For the sad-happy condition, maternal MDD history (or group), *p*=.63, or stimulus type, *p*=.34, did not influence children’s manual RT to identify the targets. There was a marginal interaction effect of group and stimulus type, *F*(1,47)=3.40, *p*=.07. For the angry-happy condition, there was a significant effect of stimulus type, *F*(1,48)=6.98, *p*=.01, such that children across both groups were faster in identifying angry than happy targets, *B*=-223.39, *SE*=87.19, *t*=-2.56, *p*=.01. no other main effects or interaction effect were significant, *p*s>.15.

**4.2. The effects of maternal MDD history and stimulus type on mothers’ RT measure**

There was a significant group difference in mothers’ RTs to identify sad and happy target faces, *F*(1,51)=5.44, *p*=.02. That is, mothers with a history of MDD were slower in identifying targets than those without a history of MDD, *B*=183.31, *SE*=80.15, *t*=2.29, *p*=.03. There was a significant effect of stimulus type, *F*(1,51)=4.89, *p*=.03. Across both groups, mothers responded faster to sad than happy targets, *B*=-68.16, *SE*=31.42, *t*=-2.17, *p*=.03. However, there was no significant interaction effect between group and stimulus type on mothers’ RTs to targets, *p*=.43. For the angry-happy condition, there was a significant group-by-stimulus interaction effect, *F*(1,50)=4.27, *p*=.04. As shown in SI Figure 2. While the model predicted RT difference between angry versus happy targets is greater in mothers with MDD history than in non-depressed mothers, both groups showed faster RT to detect angry than happy targets (MDD: *B*=-218.11, *SE*=30.68, *t*=-7.11, *p*<.001; Non-MDD: *B*=-128.49, *SE*=31.84, *t*=-4.04, *p*<.001).


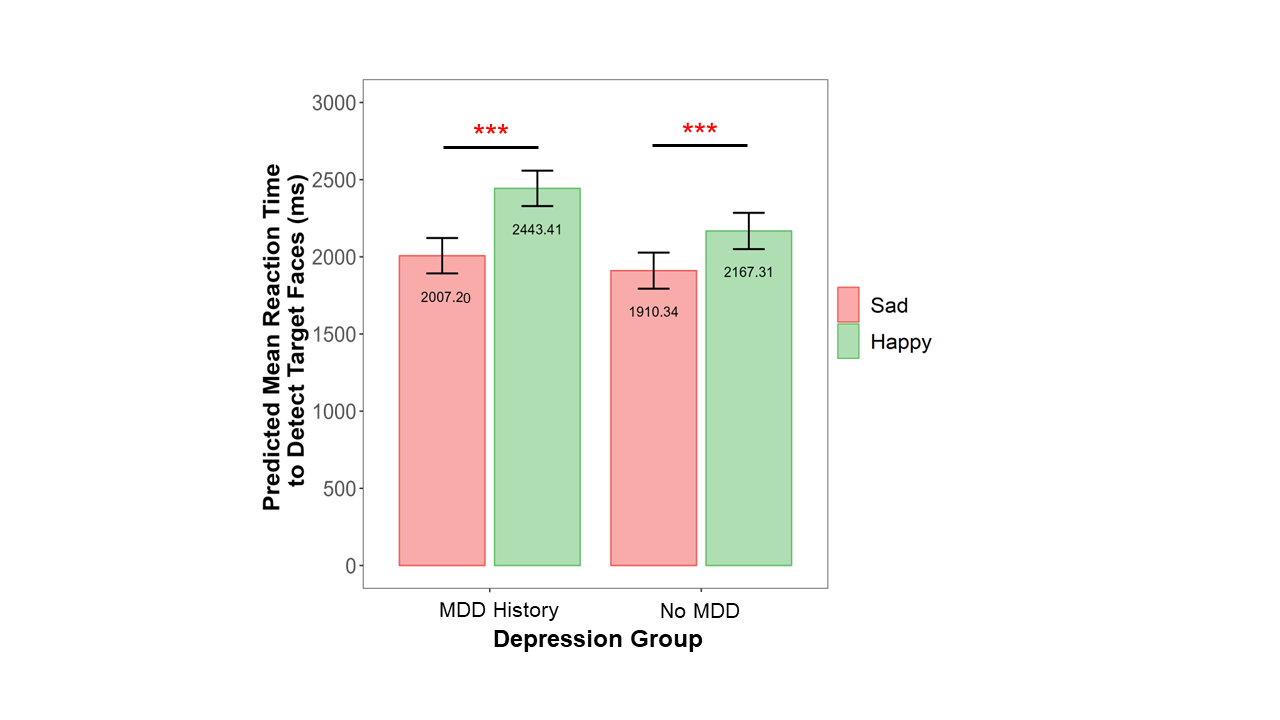


**SI Figure 2:** The interaction effect of maternal major depressive disorder history (MDD) and stimulus type (angry versus happy target faces) on mothers’ manual reaction time (RT) to response to target faces.

# 4.3. The effects of maternal depression level and stimulus type on children’s RT measure

We also examined the effect of maternal current depression level, indexed by the BDI scores, stimulus type, and their interaction effect, on the children’s RTs to identify the targets, separately for the sad-happy and angry-happy condition. For the sad-happy condition, the LME model showed that there was no significant effect of maternal depression level, *p*=.84, stimulus type, *p*=.40, or two-way interaction effect, *p*>1.00. For the angry-happy condition, there was a significant effect of stimulus type on the children’s RT to identify the targets, *F*(1,48)=6.88, *p*=.01. The effect of maternal depression level, *p*=.71, and depression-by-stimulus interaction effect was not significant, *p*=.49.

**4.4. The effects of maternal depression level and stimulus type on mothers’ RT measure**

Maternal current depression level did not impact mothers’ RTs to identify the sad and happy targets, *p*=.44. There was a significant effect of stimulus type, *F*(1,51)=4.90, *p*=.03. However, stimulus type did not moderate the effect of maternal depression level on mothers’ RTs, *p*=.90. Similarly, there was a significant effect of stimulus type for the angry-happy condition, *F*(1,50)=61.12, *p*<.001. There was no effect of maternal depression, *p*=.89 or depression-by-stimulus interaction effect, *p*=.37.

**SI Table 1**. Results from the linear mixed-effects models.

| **Parameters** | **Est.** | **95% *CI*** | ***R^2^*** |
| --- | --- | --- | --- |
| **1.1a. Outcome: mean latency of first fixation on the targets in children** | | |  |
| **Fixed effects** |  |  | 0.22 |
| Intercept | **3314.11** | (3055.26, 3572.95) |  |
| Sex | **65.22** | (-305.05, 435.49) |  |
| CBCL internalizing score | -12.60 | (-47.89, 22.69) |  |
| Maternal MDD history (Group) | 21.51 | (-167.267, 210.28) |  |
| Stimulus type (sad vs. happy) | **-257.50** | (-395.04, -119.96) |  |
| Mothers’ latency of first fixation on the targets | 0.09 | (-0.47, 0.64) |  |
| Group×Stimulus | -134.16 | (-271.705, 3.39) |  |
| Group×Mothers’ latency | -0.43 | (-0.98, 0.12) |  |
| Stimulus×Mothers’ latency | -0.38 | (-0.85, 0.09) |  |
| Group×Stimulus×Mothers’ latency | -0.27 | (-0.74, 0.20) |  |
| **Random effects** |  |  |  |
| SD Intercept | 398.13 | (229.57, 690.45) |  |
| Residual | 605.36 | (485.80, 754.33) |  |
| **1.1b. Outcome: mean latency of first fixation on the targets in children** | | |  |
| **Fixed effects** |  |  | 0.15 |
| Intercept | **3355.27** | (2995.63, 3714.92) |  |
| Sex | 285.23 | (-212.68, 783.13) |  |
| CBCL internalizing score | -28.61 | (-72.40, 15.18) |  |
| Maternal MDD history (Group) | -88.05 | (-354.50, 178.40) |  |
| Stimulus type (angry vs. happy) | **-385.95** | (-593.98, -177.91) |  |
| Mothers’ latency of first fixation on the targets | **-0.99** | (-1.89, -0.10) |  |
| Group×Stimulus | 77.14 | (-129.53, 283.81) |  |
| Group×Mothers’ latency | 0.45 | (-0.44, 1.34) |  |
| Stimulus×Mothers’ latency | -0.13 | (-0.85, 0.60) |  |
| Group×Stimulus×Mothers’ latency | 0.10 | (-0.63, 0.83) |  |
| **Random effects** |  |  |  |
| SD Intercept | 541.15 | (314.54, 931.02) |  |
| Residual | 780.26 | (621.92, 978.91) |  |
| 1.2a. Outcome: mean total dwell time on the targets in children | | |  |
| **Fixed effects** |  |  | 0.24 |
| Intercept | **1647.23** | (1413.56, 1889.90) |  |
| Sex | 248.94 | (-80.92, 578.80) |  |
| CBCL internalizing score | 8.53 | (-26.41, 43.46) |  |
| Maternal MDD history (Group) | -17.80 | (-185.01, 149.40) |  |
| Stimulus type (Sad vs. Happy) | **250.75** | (159.21, 342.29) |  |
| Mothers’ dwell time on the targets | 0.07 | (-0.01, 0.15) |  |
| Group×Stimulus | 96.40 | (4.74, 188.06) |  |
| Group×Mothers’ dwell time | -0.03 | (-0.10, 0.04) |  |
| Stimulus×Mothers’ dwell time | 0.003 | (-0.05, 0.06) |  |
| Group×Stimulus×Mothers’ dwell time | 0.02 | (-0.04, 0.07) |  |
| **Random effects** |  |  |  |
| SD Intercept | 460.99 | (330.24, 643.51) |  |
| Residual | 417.20 | (332.32, 523.77) |  |
| 1.2b. Outcome: mean total dwell time on the targets in children | | |  |
| **Fixed effects** |  |  | 0.24 |
| Intercept | **1871.97** | (1598.06, 2145.88) |  |
| Sex | -241.64 | (-632.078, 148,79) |  |
| CBCL internalizing score | 30.27 | (-9.68, 70.23) |  |
| Maternal MDD history (Group) | 36.28 | (-159.58, 232.14) |  |
| Stimulus type (Angry vs. Happy) | **363.90** | (241.32, 486.48) |  |
| Mothers’ dwell time on the targets | -0.001 | (-0.10, 0.10) |  |
| Group×Stimulus | 53.03 | (-69.55, 175.62) |  |
| Group×Mothers’ dwell time | 0.002 | (-0.09, 0.09) |  |
| Stimulus×Mothers’ dwell time | -0.03 | (-0.10, 0.04) |  |
| Group×Stimulus×Mothers’ dwell time | -0.03 | (-0.10, 0.04) |  |
| **Random effects** |  |  |  |
| SD Intercept | 478.99 | (321.36, 713.95) |  |
| Residual | 574.24 | (463.84, 710.92) |  |
| **2.1a. Outcome: mean latency of first fixation on the targets in children** | | |  |
| **Fixed effects** |  |  | 0.17 |
| Intercept | **3306.55** | (3065.29, 3547.80) |  |
| Sex | 74.71 | (-256.73, 406.15) |  |
| CBCL internalizing score | -17.79 | (-54.85, 19.28) |  |
| Maternal depression level (BDI score) | 10.39 | (-6.83, 27.61) |  |
| Stimulus type (Sad vs. Happy) | **-306.02** | (-448.74, -163.30) |  |
| BDI score×Stimulus | -7.96 | (-20.91, 5.00) |  |
| **Random effects** |  |  |  |
| SD Intercept | 289.72 | (108.27, 775.27) |  |
| Residual | 686.78 | (559.33, 843.26) |  |
| **2.1b. Outcome: mean latency of first fixation on the targets in children** | | |  |
| **Fixed effects** |  |  | 0.10 |
| Intercept | **3439.17** | (3091.51, 3786.83) |  |
| Sex | 141.13 | (-342.29, 624.55) |  |
| CBCL internalizing score | - 31.19 | (-83.69, 21.32) |  |
| Maternal depression level (BDI score) | 5.26 | (-20.25, 30.77) |  |
| Stimulus type (Angry vs. Happy) | **-264.17** | (-424.35, -103.99) |  |
| BDI score×Stimulus | 9.05 | (-5.12, 23.22) |  |
| **Random effects** |  |  |  |
| SD Intercept | 611.66 | (410.70, 910.94) |  |
| Residual | 768.08 | (625.25, 943.53) |  |
| **2.2a. Outcome: mean total dwell time on the targets in children** | | |  |
| **Fixed effects** |  |  | 0.33 |
| Intercept | **1620.59** | (1415.45, 1825.72) |  |
| Sex | 256.21 | (-25.83, 538.25) |  |
| CBCL internalizing score | **45.46** | (13.74, 77.17) |  |
| Maternal depression level (BDI score) | **-25.52** | (-40.06, -10.98) |  |
| Stimulus type (Sad vs. Happy) | **275.49** | (181.73, 369.26) |  |
| BDI score×Stimulus | **9.72** | (1.11, 18.32) |  |
| **Random effects** |  |  |  |
| SD Intercept | 368.13 | (246.18, 550.50) |  |
| Residual | 447.25 | (361.89, 552.73) |  |
| **2.2b. Outcome: mean total dwell time on the targets in children** | | |  |
| **Fixed effects** |  |  | 0.21 |
| Intercept | **1833.72** | (1572.99, 2094.46) |  |
| Sex | -181.45 | (-542.30, 179.39) |  |
| CBCL internalizing score | **45.88** | (6.51, 85.25) |  |
| Maternal depression level (BDI score) | -13.30 | (-32.38, 5.79) |  |
| Stimulus type (Angry vs. Happy) | **358.45** | (239.74, 477.17) |  |
| BDI score×Stimulus | -2.97 | (-13.46, 7.52) |  |
| **Random effects** |  |  |  |
| SD Intercept | 460.32 | (312.09, 678.95) |  |
| Residual | 573.85 | (468.58, 702.77) |  |
| **2.3a. Outcome: mean latency of first fixation on the targets in mothers** | | |  |
| **Fixed effects** |  |  | 0.02 |
| Intercept | **1361.23** | (1287.93, 1434.52) |  |
| Maternal depression level (BDI score) | 1.44 | (-4.93, 7.81) |  |
| Stimulus type (Sad vs. Happy) | -38.02 | (-80.29, 4.24) |  |
| BDI score×Stimulus | 0.34 | (-3.40, 4.08) |  |
| **Random effects** |  |  |  |
| SD Intercept | 217.46 | (159.41, 296.64) |  |
| Residual | 211.43 | (173.04, 258.33) |  |
| **2.3b. Outcome: mean latency of first fixation on the targets in mothers** | | |  |
| **Fixed effects** |  |  | 0.21 |
| Intercept | **1337.21** | (1278.16, 1396.25) |  |
| Maternal depression level (BDI score) | 0.74 | (-4.35, 5.82) |  |
| Stimulus type (Angry vs. Happy) | -123.82 | (-157.09, 90.55) |  |
| BDI score×Stimulus | 0.28 | (-2.58, 3.14) |  |
| **Random effects** |  |  |  |
| SD Intercept | 176.92 | (132.27, 236.66) |  |
| Residual | 169.30 | (139.80, 205.02) |  |
| **2.4a. Outcome: mean total dwell time on the targets in mothers** | | |  |
| **Fixed effects** |  |  | 0.06 |
| Intercept | **3672.38** | (3199.79, 4144.97) |  |
| Maternal depression level (BDI score) | 33.58 | (-7.50, 74.66) |  |
| Stimulus type (Sad vs. Happy) | 203.82 | (-24.39, 432.04) |  |
| BDI score×Stimulus | 15.52 | (-4.86, 35.90) |  |
| **Random effects** |  |  |  |
| SD Intercept | 1500.91 | (1163.31, 1936.49) |  |
| Residual | 1159.75 | (956.97, 1405.49) |  |
| **2.4b. Outcome: mean total dwell time on the targets in mothers** | | |  |
| **Fixed effects** |  |  | 0.05 |
| Intercept | **3826.54** | (3344.94, 4308.14) |  |
| Maternal depression level (BDI score) | 36.57 | (-4.92, 78.05) |  |
| Stimulus type (Angry vs. Happy) | 40.53 | (-140.86, 221.93) |  |
| BDI score×Stimulus | 3.06 | (-12.51, 18.64) |  |
| **Random effects** |  |  |  |
| SD Intercept | 1617.94 | (1293.95, 2023.04) |  |
| Residual | 921.98 | (761.08, 1116.89) |  |
| **3.1a. Outcome: mean latency of first fixation on the targets in children** | | |  |
| **Fixed effects** |  |  | 0.22 |
| Intercept | **3275.73** | (3024.58, 3526.88) |  |
| Sex | 120.49 | (-235.44, 476.42) |  |
| CBCL internalizing score | -23.07 | (-63.88, 17.74) |  |
| Maternal depression level (BDI score) | 3.94 | (-14.86, 22.75) |  |
| Stimulus type (Sad vs. Happy) | **-278.74** | (-413.59, - 143.89) |  |
| Mothers’ latency of first fixation on the targets | 0.05 | (-0.51, 0.61) |  |
| BDI score×Stimulus | -6.88 | (-19.80, 6.04) |  |
| BDI score×Mothers’ latency | **-0.07** | (-0.12, -0.01) |  |
| Stimulus×Mothers’ latency | -0.34 | (-0.81, 0.13) |  |
| BDI score ×Stimulus×Mothers’ latency | -0.02 | (-0.06, 0.03) |  |
| **Random effects** |  |  |  |
| SD Intercept | 382.51 | (206.14, 709.77) |  |
| Residual | 607.05 | (483.34, 762.43) |  |
| **3.1b. Outcome: mean latency of first fixation on the targets in children** | | |  |
| **Fixed effects** |  |  | 0.15 |
| Intercept | **3373.61** | (3011.53, 3735.70) |  |
| Sex | 263.86 | (-230.45, 758.17) |  |
| CBCL internalizing score | -42.26 | (-95.96, 11.45) |  |
| Maternal depression level (BDI score) | 4.46 | (-22.65, 31.57) |  |
| Stimulus type (Angry vs. Happy) | **-388.48** | (-589.35, -187.61) |  |
| Mothers’ latency of first fixation on the targets | **-1.04** | (-1.94, -0.13) |  |
| BDI score×Stimulus | 8.81 | (-9.22, 26.84) |  |
| BDI score×Mothers’ latency | 0.01 | (-0.08, 0.10) |  |
| Stimulus×Mothers’ latency | -0.12 | (-0.81, 0.57) |  |
| BDI score ×Stimulus×Mothers’ latency | -0.03 | (-0.10, 0.05) |  |
| **Random effects** |  |  |  |
| SD Intercept | 621.41 | (415.09, 930.28) |  |
| Residual | 734.89 | (590.79, 914.14) |  |
| **3.2a. Outcome: mean total dwell time on the targets in children** | | |  |
| **Fixed effects** |  |  | 0.35 |
| Intercept | **1604.48** | (1398.86, 1810.10) |  |
| Sex | 276.08 | (-7.66, 559.82) |  |
| CBCL internalizing score | 29.64 | (-5.59, 64.87) |  |
| Maternal depression level (BDI score) | **-24.89** | (-39.80, -9.98) |  |
| Stimulus type (Sad vs. Happy) | **252.01** | (155.45, 348.56) |  |
| Mothers’ dwell time on the targets | 0.07 | (-0.01, 0.14) |  |
| BDI score ×Stimulus | 7.81 | (-1.48, 17.10) |  |
| BDI score ×Mothers’ dwell time | 0.004 | (-0.002, 0.01) |  |
| Stimulus×Mothers’ dwell time | 0.0004 | (-0.05, 0.05) |  |
| Group×Stimulus×Mothers’ dwell time | -0.0002 | (-0.01, 0.004) |  |
| **Random effects** |  |  |  |
| SD Intercept | 365.27 | (235.21, 567.23) |  |
| Residual | 439.48 | (349.77, 552.21) |  |
| **3.2b. Outcome: mean total dwell time on the targets in children** | | |  |
| **Fixed effects** |  |  | 0.27 |
| Intercept | **1573.96** | (1227.11, 1920.81) |  |
| Sex | -191.89 | (-557.10, 173.31) |  |
| CBCL internalizing score | 43.82 | (0.30, 87.34) |  |
| Maternal depression level (BDI score) | **-**16.12 | (-35.70, 3.46) |  |
| Stimulus type (Angry vs. Happy) | **353.06** | (225.61, 480.52) |  |
| Mothers’ dwell time on the targets | -0.002 | (-0.10, 0.10) |  |
| BDI score ×Stimulus | -1.36 | (-13.26, 10.55) |  |
| BDI score ×Mothers’ dwell time | 0.005 | (-0.002, 0.01) |  |
| Stimulus×Mothers’ dwell time | -0.03 | (-0.10, 0.04) |  |
| Group×Stimulus×Mothers’ dwell time | -0.0004 | (-0.006, 0.005) |  |
| **Random effects** |  |  |  |
| SD Intercept | 444.46 | (287.50, 687.11) |  |
| Residual | 583.04 | (471.81, 720.51) |  |
| **4.1a. Outcome: mean manual reaction time on the targets in children** | | |  |
| **Fixed effects** |  |  | 0.06 |
| Intercept | **5637.60** | (5181.84, 6093.36) |  |
| Sex | -445.98 | (-1082.96, 191.00) |  |
| CBCL internalizing score | 37.10 | (-24.98, 99.18) |  |
| Maternal MDD history (Group) | 76.86 | (-244.51, 398.22) |  |
| Stimulus type (Sad vs. Happy) | -58.46 | (-181.40, 64.48) |  |
| Group×Stimulus | -112.70 | (-235.66, 10.26) |  |
| **Random effects** |  |  |  |
| SD Intercept | 1009.04 | (800.93, 1271.23) |  |
| Residual | 607.19 | (498.41, 739.71) |  |
| **4.1b. Outcome: mean manual reaction time on the targets in children** | | |  |
| **Fixed effects** |  |  | 0.06 |
| Intercept | **5442.96** | (4955.19, 5930.73) |  |
| Sex | -314.43 | (-994.31, 365.46) |  |
| CBCL internalizing score | -1.17 | (-62.56, 60.22) |  |
| Maternal MDD history (Group) | 117.18 | (-226.51, 460.87) |  |
| Stimulus type (Angry vs. Happy) | **-223.39** | (-393.36, -53.42) |  |
| Group×Stimulus | 123.97 | (-46.00, 293.94) |  |
| **Random effects** |  |  |  |
| SD Intercept | 943.47 | (709.11, 1255.30) |  |
| Residual | 844.68 | (694.34, 1027.57) |  |
| **4.2a. Outcome: mean manual reaction time on the targets in mothers** | | |  |
| **Fixed effects** |  |  | 0.09 |
| Intercept | **2069.61** | (1911.76, 2227.46) |  |
| Maternal MDD history (Group) | **183.31** | (25.47, 341.16) |  |
| Stimulus type (Sad vs. Happy) | **-68.16** | (-130.05, -6.28) |  |
| Group×Stimulus | -24.33 | (-86.22, 37.55) |  |
| **Random effects** |  |  |  |
| SD Intercept | 526.48 | (419.32, 661.03) |  |
| Residual | 317.31 | (262.31, 383.85) |  |
| **4.2b. Outcome: mean manual reaction time on the targets in mothers** | | |  |
| **Fixed effects** |  |  | 0.11 |
| Intercept | **2132.07** | (1973.41, 2290.72) |  |
| Maternal MDD history (Group) | 93.24 | (-65.42, 251.90) |  |
| Stimulus type (Angry vs. Happy) | **-173.30** | (-216.85, -129.75) |  |
| Group×Stimulus | **-44.81** | (-88.36, -1.26) |  |
| **Random effects** |  |  |  |
| SD Intercept | 553.16 | (450.12, 679.79) |  |
| Residual | 221.11 | (182.49, 267.89) |  |
| **4.3a. Outcome: mean manual reaction time on the targets in children** | | |  |
| **Fixed effects** |  |  | 0.05 |
| Intercept | **5630.57** | (5178.11, 6083.02) |  |
| Sex | -429.31 | (-1055.88, 197.26) |  |
| CBCL internalizing score | 35.83 | (-35.42, 107.08) |  |
| Maternal depression level (BDI score) | 3.55 | (-29.86, 36.96) |  |
| Stimulus type (Sad vs. Happy) | - 55.79 | (-182.78, 71.21) |  |
| BDI score×Stimulus | -0.01 | (-11.60, 11.59) |  |
| **Random effects** |  |  |  |
| SD Intercept | 1007.73 | (797.63, 1273.15) |  |
| Residual | 626.59 | (514.34, 763.34) |  |
| **4.3b. Outcome: mean manual reaction time on the targets in children** | | |  |
| **Fixed effects** |  |  | 0.05 |
| Intercept | **5630.57** | (5178.11, 6083.02) |  |
| Sex | -429.31 | (-1055.88, 197.26) |  |
| CBCL internalizing score | 35.83 | (-35.42, 107.08) |  |
| Maternal depression level (BDI score) | 3.55 | (-29.86, 36.96) |  |
| Stimulus type (Angry vs. Happy) | - 55.79 | (-182.78, 71.21) |  |
| BDI score×Stimulus | -0.01 | (-11.60, 11.59) |  |
| **Random effects** |  |  |  |
| SD Intercept | 1007.73 | (797.63, 1273.15) |  |
| Residual | 626.59 | (514.34, 763.34) |  |
| **4.4a. Outcome: mean manual reaction time on the targets in mothers** | | |  |
| **Fixed effects** |  |  | 0.02 |
| Intercept | **2073.07** | (1908.31, 2237.83) |  |
| Maternal depression level (BDI score) | 5.55 | (-8.65, 19.75) |  |
| Stimulus type (Sad vs. Happy) | **-68.62** | (-130.85, -6.40) |  |
| BDI score×Stimulus | 0.34 | (-5.03, 5.70) |  |
| **Random effects** |  |  |  |
| SD Intercept | 553.22 | (442.07, 692.32) |  |
| Residual | 319.12 | (263.80, 386.04) |  |
| **4.4b. Outcome: mean manual reaction time on the targets in mothers** | | |  |
| **Fixed effects** |  |  | 0.08 |
| Intercept | **2134.50** | (1973.86, 2295.14) |  |
| Maternal depression level (BDI score) | 1.00 | (-12.84, 14.84) |  |
| Stimulus type (Angry vs. Happy) | **-174.82** | (-219.73, -129.90) |  |
| BDI score×Stimulus | -1.75 | (-5.60, 2.11) |  |
| **Random effects** |  |  |  |
| SD Intercept | 559.32 | (454.82, 687.83) |  |
| Residual | 228.17 | (188.32, 276.46) |  |

*Note*: Est.=beta values. CI=Confidence interval. Estimates are bolded if their 95% CI does not contain zero, suggesting a significant effect. *p* values are reported in the text. BDI = Beck Depression Inventory

**SI Table 2**. Mean and (standard deviations) for the reaction time (RT) to identify the target faces in children and their mothers.

|  | Depressed Mothers  (High-Risk Children) | Never-Depressed Mothers  (Low-Risk Children) |
| --- | --- | --- |
| RT Angry target | 5236.21(1133.03) | 4858.93(1611.43) |
| RT Happy target in HA trials | 5435.04(1176.07) | 5553.65(1205.49) |
| RT Sad target | 5628.90(1141.59) | 5226.74(1315.37) |
| RT Happy target in HS trials | 5301.88(1005.95) | 5404.70(1412.21) |

**SI Reference**

Beck, A. T., Steer, R. A., & Brown, G. K. (1996). *Manual for the Beck Depression Inventory-II*. San Antonio: Psychological Corporation.
